# Persistent COVID-19 Symptoms at 6 Months After Onset and the Role of Vaccination Before or After SARS-CoV-2 Infection

**DOI:** 10.1001/jamanetworkopen.2022.51360

**Published:** 2023-01-18

**Authors:** Stephanie A. Richard, Simon D. Pollett, Anthony C. Fries, Catherine M. Berjohn, Ryan C. Maves, Tahaniyat Lalani, Alfred G. Smith, Rupal M. Mody, Anuradha Ganesan, Rhonda E. Colombo, David A. Lindholm, Michael J. Morris, Nikhil Huprikar, Christopher J. Colombo, Cristian Madar, Milissa Jones, Derek T. Larson, Samantha E. Bazan, Katrin Mende, David Saunders, Jeffrey Livezey, Charlotte A. Lanteri, Ann I. Scher, Celia Byrne, Jennifer Rusiecki, Evan Ewers, Nusrat J. Epsi, Julia S. Rozman, Caroline English, Mark P. Simons, David R. Tribble, Brian K. Agan, Timothy H. Burgess

**Affiliations:** 1 Infectious Disease Clinical Research Program, Department of Preventive Medicine and Biostatistics, Uniformed Services University of the Health Sciences, Bethesda, Maryland; 2 The Henry M. Jackson Foundation for the Advancement of Military Medicine, Inc, Bethesda, Maryland; 3 US Air Force School of Aerospace Medicine, Dayton, Ohio; 4 Naval Medical Center San Diego, San Diego, California; 5 Department of Medicine, Uniformed Services University of the Health Sciences, Bethesda, Maryland; 6 Naval Medical Center Portsmouth, Portsmouth, Virginia; 7 William Beaumont Army Medical Center, Fort Bliss, Texas; 8 Walter Reed National Military Medical Center, Bethesda, Maryland; 9 Madigan Army Medical Center, Joint Base Lewis-McChord, Washington; 10 Brooke Army Medical Center, Joint Base San Antonio–Fort Sam Houston, Texas; 11 Tripler Army Medical Center, Honolulu, Hawaii; 12 Department of Pediatrics, Uniformed Services University of the Health Sciences, Bethesda, Maryland; 13 Fort Belvoir Community Hospital, Fort Belvoir, Virginia; 14 Carl R. Darnall Army Medical Center, Fort Hood, Texas; 15 Department of Preventive Medicine and Biostatistics, Uniformed Services University of the Health Sciences, Bethesda, Maryland

## Abstract

**Importance:**

Understanding the factors associated with post-COVID conditions is important for prevention.

**Objective:**

To identify characteristics associated with persistent post–COVID-19 symptoms and to describe post–COVID-19 medical encounters.

**Design, Setting, and Participants:**

This cohort study used data from the Epidemiology, Immunology, and Clinical Characteristics of Emerging Infectious Diseases With Pandemic Potential (EPICC) study implemented in the US military health system (MHS); MHS beneficiaries aged 18 years or older who tested positive for SARS-CoV-2 from February 28, 2020, through December 31, 2021, were analyzed, with 1-year follow-up.

**Exposures:**

SARS-CoV-2 infection.

**Main Outcomes and Measures:**

The outcomes analyzed included survey-reported symptoms through 6 months after SARS-CoV-2 infection and *International Statistical Classification of Diseases and Related Health Problems, Tenth Revision* diagnosis categories reported in medical records 6 months following SARS-CoV-2 infection vs 3 months before infection.

**Results:**

More than half of the 1832 participants in these analyses were aged 18 to 44 years (1226 [66.9%]; mean [SD] age, 40.5 [13.7] years), were male (1118 [61.0%]), were unvaccinated at the time of their infection (1413 [77.1%]), and had no comorbidities (1290 [70.4%]). A total of 728 participants (39.7%) had illness that lasted 28 days or longer (28-89 days: 364 [19.9%]; ≥90 days: 364 [19.9%]). Participants who were unvaccinated prior to infection (risk ratio [RR], 1.39; 95% CI, 1.04-1.85), reported moderate (RR, 1.80; 95% CI, 1.47-2.22) or severe (RR, 2.25; 95% CI, 1.80-2.81) initial illnesses, had more hospitalized days (RR per each day of hospitalization, 1.02; 95% CI, 1.00-1.03), and had a Charlson Comorbidity Index score of 5 or greater (RR, 1.55; 95% CI, 1.01-2.37) were more likely to report 28 or more days of symptoms. Among unvaccinated participants, postinfection vaccination was associated with a 41% lower risk of reporting symptoms at 6 months (RR, 0.59; 95% CI, 0.40-0.89). Participants had higher risk of pulmonary (RR, 2.00; 95% CI, 1.40-2.84), diabetes (RR, 1.46; 95% CI, 1.00-2.13), neurological (RR, 1.29; 95% CI, 1.02-1.64), and mental health–related medical encounters (RR, 1.28; 95% CI, 1.01-1.62) at 6 months after symptom onset than at baseline (before SARS-CoV-2 infection).

**Conclusions and Relevance:**

In this cohort study, more severe acute illness, a higher Charlson Comorbidity Index score, and being unvaccinated were associated with a higher risk of reporting COVID-19 symptoms lasting 28 days or more. Participants with COVID-19 were more likely to seek medical care for diabetes, pulmonary, neurological, and mental health–related illness for at least 6 months after onset compared with their pre-COVID baseline health care use patterns. These findings may inform the risk-benefit ratio of COVID-19 vaccination policy.

## Key Points

**Question:**

What factors are associated with persistent post–COVID-19 symptoms, and how do post–COVID-19 medical encounters change over time?

**Findings:**

In this cohort study of 1832 US adults, the risk of reporting symptoms for 28 or more days after COVID-19 onset was significantly higher in participants who were unvaccinated at the time of infection and those who reported moderate or severe acute illness symptoms. At 6 months after onset, participants had significantly higher risk of pulmonary, diabetes, neurological, and mental health encounters vs preinfection baseline.

**Meaning:**

The findings suggest that COVID-19 is associated with increased health care encounters through 6 months after infection; vaccination was associated with lower risk of long-term COVID-19 symptoms.

## Teaser

This cohort study investigates factors associated with COVID-19 symptoms in the 6 months after infection and compares the frequency of medical encounters before and after infection.

## Introduction

Infection with SARS-CoV-2 can result in a variety of acute outcomes ranging from asymptomatic infection or minor symptoms to hospitalization and death.^1^ Our understanding of the diverse array of acute clinical phenotypes and patient-reported outcomes and the effect of countermeasures has substantially improved even as new variants have emerged. In early 2020, it was noted that patients with COVID-19 frequently reported symptoms that persisted for months after infection, now generally described as post–COVID-19 conditions (PCCs) but colloquially known as “long COVID”^2,3,4,5,6,7,8,9,10^; PCCs have been associated with fatigue, shortness of breath, joint pain, anxiety, depression, and other symptoms.^2,3,4,5,6,7,8,9,10^ The duration, clinical phenotype(s), and pathophysiology of PCCs are still being defined and may vary with different viral variants. It is important to evaluate the risk and risk factors for PCCs in a range of populations, including younger, healthier populations who may be at lower risk of severe acute illness.

Data from clinical trials and observational studies have demonstrated that vaccination against SARS-CoV-2 is highly protective against acute symptoms, hospitalization, and death from COVID-19 across a range of variants.^11,12,13,14^ The relationship between SARS-CoV-2 vaccination and PCCs remains unclear,^15,16,17^ and some studies suggest that vaccination after infection may mitigate the risk of PCCs.^15,18,19^ If vaccination either before or after infection decreases the impact of PCCs, there are even stronger arguments for vaccination. Therefore, further evidence is needed urgently to better clarify the role of vaccination as a potential intervention for PCCs.

The Epidemiology, Immunology, and Clinical Characteristics of Emerging Infectious Diseases With Pandemic Potential (EPICC) study is a longitudinal observational cohort study of US Military Health System (MHS) beneficiaries that aims to describe clinical outcomes of SARS-CoV-2 infection, including long-term sequelae.^20^ In this analysis, we (1) described long-term experiences after COVID-19 and factors associated with risk for PCCs in the EPICC study cohort, (2) quantified the association of vaccination before infection and vaccination after infection with persistent COVID-19 symptoms, and (3) compared the frequency of organ-specific health care encounters before and after SARS-CoV-2 infection.

## Methods

### Ethics

This cohort study used data from the EPICC study, which was approved by the Uniformed Services University institutional review board; all study participants provided written informed consent when enrolled. This study was conducted following good clinical practice and according to the Declaration of Helsinki.^21^ The investigators adhered to the policies for protection of human participants as prescribed in 45 CFR §46. We followed the Strengthening the Reporting of Observational Studies in Epidemiology (STROBE) reporting guideline.

### Study Population and General Study Design

The EPICC study cohort has been described in detail elsewhere.^20^ In brief, MHS beneficiaries (active duty, dependents, and retirees) were recruited into the EPICC study via in-person and online pathways. Eligibility for enrollment into the EPICC study included testing for SARS-CoV-2, presentation with a COVID-19–like illness, and/or exposure to a known COVID-19 case, and in early 2021, eligibility was expanded to include receipt of a SARS-CoV-2 vaccine. The EPICC study began in March 2020, with participants actively followed up for 1 year with collection of demographics, medical history, illness and risk factor data, and biological specimens at various times during their follow-up (eTable 1 in Supplement 1). The participants included in the current study’s analysis had a COVID-19 symptom onset date (or date first positive for SARS-CoV-2) through December 31, 2021, to allow for 6 months of MHS data accumulation.

### SARS-CoV-2 Infection Case Diagnosis Criteria

The EPICC study participants included in these analyses were adults (age 18 years or older) who tested positive for SARS-CoV-2 and had complete demographic data available (eFigure 1 in Supplement 1). SARS-CoV-2–positive status was identified using medical records (positive clinical polymerase chain reaction test result), surveys (participant report of a positive nose or throat swab test result), and study-collected swab samples.^20^ For these analyses, we categorized time into a pre-Delta period (March 1, 2020, through June 30, 2021) and a Delta period (July 1 through December 31, 2021), estimated using US variant data from Covariants/Global Initiative on Sharing Avian Influenza Data.^22^ We excluded participants (n = 549) with suspected reinfections (defined here as positive SARS-CoV-2 test results more than 90 days apart) because symptoms that they reported after the primary infection might have been related to a reinfection.

### Demographic and Clinical Characteristics

Study staff collected demographic and clinical characteristics using standardized case report forms for participants enrolled at a military treatment facility, and starting in November 2020, all participants completed periodic online surveys (enrollment and at 1, 3, 6, 9, and 12 months) that ascertained their experience with SARS-CoV-2 infection. Participants who had enrolled before November 2020 completed a catch-up survey with similar questions and then completed the remaining surveys according to their time in the study. Participants who reported COVID-19 symptoms were asked about the date of symptom onset and the duration and severity of the illness or symptoms. Specifically, participants were asked to rate their overall illness and symptoms at their worst with the options of “never had symptoms,” “mild (noticeable but not impairing),” “moderate (impairing but not disabling; interferes with duties),” “severe (disabling; can’t perform duties),” and “critical (life threatening).” Participants were considered to have been hospitalized for COVID-19 if they reported hospitalization due to COVID-19 on a survey or if the study staff reported that the participant was hospitalized due to COVID-19 based on a review of the medical record (military treatment facility participants only). Participants reported the number of days they were hospitalized; for inclusion into the model, individuals with no hospitalization history were recorded as having no days of hospitalization.

If participants reported symptoms that lasted for more than a month, they were asked about specific symptom presence (eg, cough, wheezing) and severity using a similar severity rating as used for the overall severity. For an analysis of the effects of postonset vaccination, we retained the surveys closest to 1, 3, 6, 9, and 12 months after symptom onset (or date of the first positive SARS-CoV-2 test result if the onset date was unavailable). We excluded those who had been vaccinated within 2 weeks prior to the survey date because vaccine reactogenicity symptoms may overlap with symptoms of COVID-19. We calculated the Charlson Comorbidity Index (CCI)^23^ score using comorbidities identified in the MHS Data Repository (MDR) during the year prior to COVID-19 symptom onset. Participants were considered fully vaccinated if they received their final dose (first dose for Ad26.COV2.S and second dose for the mRNA vaccines [mRNA-1273, BNT162b2]) 14 or more days prior to onset of symptoms or the date of the first positive SARS-CoV-2 test result.

### Participant-Reported Outcomes

Participants’ survey data were summarized to describe the status of the illness (resolved or ongoing), the duration of illness, and the prevalence of longer-term symptoms. An illness was considered to be resolved if the participant did not report COVID-19 symptoms on 2 consecutive surveys or if they did not report symptoms on the last survey they completed and ongoing if they reported COVID-19 symptoms on the last survey they completed. If a participant reported cough, wheezing, or difficulty breathing during the acute phase of the illness, they were categorized as having acute respiratory symptoms.

### Medical Encounters Before and After SARS-CoV-2 Infection

As part of the informed consent process, participants consented to allow researchers to access their electronic medical record history using the MDR. Diagnoses noted during the medical encounter were grouped by organ systems using *International Statistical Classification of Diseases and Related Health Problems, Tenth Revision* codes defined in eTable 2 in Supplement 1.^20^

### Statistical Analysis

We ran Poisson regression models to identify factors associated with long (≥28 and ≥90 days) duration of COVID-19, considering demographic characteristics (age, sex), vaccination status at the time of illness onset, infection during the Delta variant period (July 1 to December 31, 2021), maximum reported COVID-19 severity, number of days hospitalized for COVID-19, acute respiratory symptoms, comorbidities, and body mass index (BMI).

As participants could be enrolled at any point prior to or following the onset of their symptoms (or first positive SARS-CoV-2 test result), we categorized the time after infection into different groups and identified whether a participant reported symptoms at that time. To assess the effects of vaccination after infection, we retained those who were unvaccinated at the time of COVID-19 symptom onset; in that subset, we conducted separate Poisson regression models to calculate the risk of reporting COVID-19 symptoms at 1, 3, 6, 9, and 12 months after symptom onset.

The proportion of participants with medical encounter–based diagnoses in each organ system on each day relative to COVID-19 symptom onset was calculated from 90 days prior to onset to 6 months after symptom onset. For the Poisson regression model, data were summarized into 30-day periods before and after symptom onset, and if a person had at least 1 medical encounter in an organ system category during that 30-day period, they were considered positive for that organ system diagnosis during that period. Separate models were fit using each organ system category as the outcome, and the models included time in 30-day periods around symptom onset, age, sex, COVID-19 severity, vaccination status, Delta period, and BMI category, as well as a random effect for the participant. The models calculated the risk of each diagnosis category in each month relative to the participant’s baseline diagnoses.

All analyses were conducted in R, version 4.1.2 (R Project for Statistical Computing). Two-sided *P* < .05 was considered significant.

## Results

**Figure 1.**
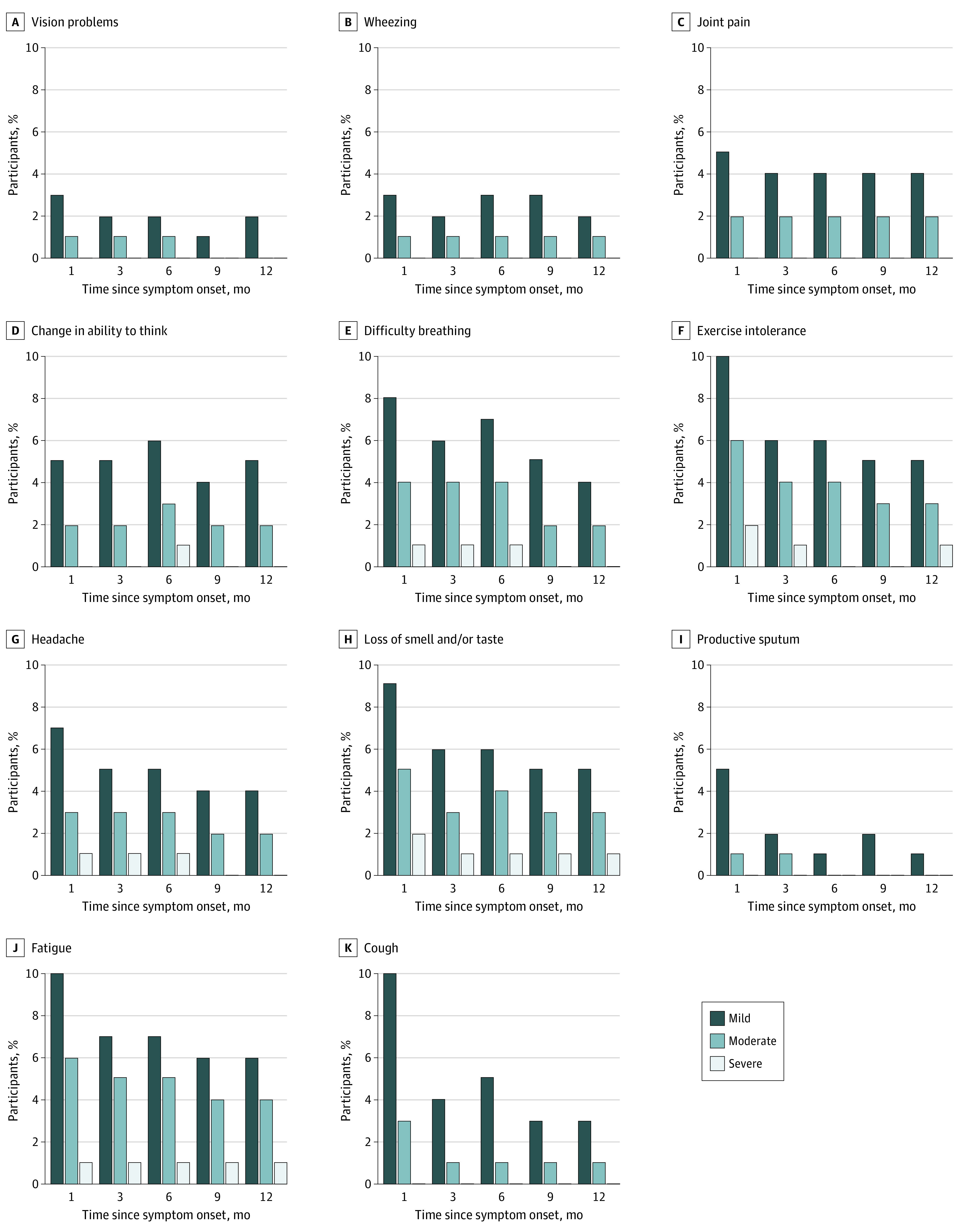
Percentage of Epidemiology, Immunology, and Clinical Characteristics of Emerging Infectious Diseases With Pandemic Potential (EPICC) Study Participants Who Endorsed Specific Symptoms on Surveys Conducted at 1, 3, 6, 9, and 12 Months

Among the 3663 participants positive for SARS-CoV-2 with onset or date of first positive SARS-CoV-2 test result between February 28, 2020, and December 31, 2021, 1832 (50.0%) were adults who completed surveys more than 14 days after symptom onset, had complete demographic data, and did not have suspected reinfections (eFigure 1 in Supplement 1). The 1832 participants included in this study were primarily 18 to 44 years of age (1226 [66.9%]; mean [SD] age, 40.5 [13.7] years) and male (1118 [61.0%] vs 714 [39.0%] female), had no marked comorbidities (1290 [70.4%]), and were unvaccinated at the time of SARS-CoV-2 infection (1413 [77.1%]) (Table 1). Only 236 participants (12.9%) were hospitalized due to COVID-19. A total of 728 participants (39.7%) had illness that lasted 28 days or longer (28-89 days: 364 [19.9%]; ≥90 days: 364 [19.9%]). Among these participants, the most common symptoms that were rated moderate or severe 1 month after symptom onset were fatigue (47 [6.5%]), exercise intolerance (43 [5.9%]), difficulty breathing (34 [4.7%]), loss of sense of smell and/or taste (39 [5.3%]), and cough (28 [3.8%]) (Figure 1). Among those who filled out a 6-month survey (1138 [62.1%]), 111 (9.8%) reported currently having a COVID-19–related symptom.

**Table 1.** Characteristics of EPICC Study Participants Included in Analyses

Characteristic	Participants, No. (%)			*P* value^a^
	Unvaccinated at time of infection (n = 1413)	Postvaccination infection (n = 419)	Total (N = 1832)	
Age group, y				
18-44	916 (64.8)	310 (74.0)	1226 (66.9)	.002
45-64	398 (28.2)	89 (21.2)	487 (26.6)	
≥65	99 (7.0)	20 (4.8)	119 (6.5)	
Sex				
Female	563 (39.8)	151 (36.0)	714 (39.0)	.16
Male	850 (60.2)	268 (64.0)	1118 (61.0)	
Race and ethnicity^b^				
Asian	68 (4.8)	10 (2.4)	78 (4.3)	<.001
Black or African American	164 (11.6)	42 (10.0)	206 (11.2)	
Hispanic or Latinx	326 (23.1)	58 (13.8)	384 (21.0)	
White	737 (52.2)	273 (65.2)	1010 (55.1)	
Other or missing^c^	118 (8.4)	36 (8.6)	154 (8.4)	
BMI category^d^				
Underweight or normal weight	292 (20.7)	107 (25.5)	399 (21.8)	.003
Overweight	577 (40.8)	183 (43.7)	760 (41.5)	
Obesity	338 (23.9)	94 (22.4)	432 (23.6)	
Severe obesity	206 (14.6)	35 (8.4)	241 (13.2)	
Charlson Comorbidity Index score				
0	954 (67.5)	336 (80.2)	1290 (70.4)	<.001
1-2	315 (22.3)	58 (13.8)	373 (20.4)	
3-4	85 (6.0)	17 (4.1)	102 (5.6)	
≥5	59 (4.2)	8 (1.9)	67 (3.7)	
Military status				
Active duty	817 (57.8)	309 (73.7)	1126 (61.5)	<.001
Dependent	316 (22.4)	67 (16.0)	383 (20.9)	
Retired military	280 (19.8)	43 (10.3)	323 (17.6)	
Infected during Delta period^e^	111 (7.9)	373 (89.0)	484 (26.4)	<.001
Fully vaccinated after SARS-CoV-2 infection				
No	246 (17.4)	419 (100.0)	665 (36.3)	<.001
Yes	1167 (82.6)	0 (0.0)	1167 (63.7)	
Hospitalization status				
Hospitalized	223 (15.8)	13 (3.1)	236 (12.9)	<.001
Not hospitalized	1190 (84.2)	406 (96.9)	1596 (87.1)	
Duration of hospitalization for COVID-19, median (IQR), d	5.0 (3.0-8.0)	3.5 (2.2-6.2)	5.0 (3.0-8.0)	.15^f^
Maximum reported severity on survey				
Never had symptoms	199 (14.1)	18 (4.3)	217 (11.8)	<.001
Mild	309 (21.9)	72 (17.2)	381 (20.8)	
Moderate	534 (37.8)	200 (47.7)	734 (40.1)	
Severe	336 (23.8)	125 (29.8)	461 (25.2)	
Critical	35 (2.5)	4 (1.0)	39 (2.1)	
Reported recovery from COVID-19				
No	121 (8.6)	23 (5.5)	144 (7.9)	.04
Yes	1292 (91.4)	396 (94.5)	1688 (92.1)	
Duration of resolved illnesses				
Median (IQR), d	16.0 (7.0-38.2)	14.0 (5.0-28.2)	15.0 (7.0-33.0)	<.001^f^
Missing data, No.	121	23	144	NA
Duration of ongoing illnesses, d				
Median (IQR)	353.0 (219.0-375.0)	276.0 (205.5-353.5)	351.5 (218.8-372.0)	.04^f^
Missing data, No.	1292	396	1688	NA
Duration group, d				
<28	811 (57.4)	293 (69.9)	1104 (60.3)	<.001
28-89	289 (20.5)	75 (17.9)	364 (19.9)	
≥90	313 (22.2)	51 (12.2)	364 (19.9)	
				
Any longer-term symptoms reported^g^				
No	1126 (79.7)	353 (84.2)	1479 (80.7)	.04
Yes	287 (20.3)	66 (15.8)	353 (19.3)	
Any moderate to severe longer-term symptoms reported^g^				
No	1184 (83.8)	369 (88.1)	1553 (84.8)	.03
Yes	229 (16.2)	50 (11.9)	279 (15.2)	
Reported respiratory symptoms on enrollment survey				
No	902 (63.8)	212 (50.6)	1114 (60.8)	<.001
Yes	511 (36.2)	207 (49.4)	718 (39.2)	

^a^ Statistical comparisons used the Pearson χ^2^ test, unless otherwise specified.

^b^ Ascertained by survey self-report and included in the analysis because racial and ethnic minorities have been shown to experience more severe short-term outcomes of SARS-CoV-2 infection.

^c^ Other race and ethnicity includes those reporting multiple races and ethnicities as well as Native American individuals.

^d^ Underweight or normal weight was defined as less than 25; overweight, 25 to 29; obesity, 30 to 34; and severe obesity, 35 or higher.

^e^ July 1 to December 31, 2021.

^f^ Kruskal-Wallis rank sum test.

^g^ Lasting more than 1 month.

We calculated the risk of reporting 28 or more and 90 or more days of COVID-19 symptoms (Table 2). Participants who were unvaccinated at the time of infection (risk ratio [RR], 1.39; 95% CI, 1.04-1.85) and reported moderate or severe initial illnesses (moderate: RR, 1.80; 95% CI, 1.47-2.22; severe: RR, 2.25; 95% CI, 1.80-2.81), more days of hospitalization (RR per each day of hospitalization, 1.02; 95% CI, 1.00-1.03), and CCI scores of 5 or higher (RR, 1.55; 95% CI, 1.01-2.37) were more likely to report 28 or more days of symptoms. When considering the outcome of 90 or more days of symptoms, the results were similar; however, vaccination and CCI scores of 5 or higher were no longer associated with the outcome. There was no association between primary infection during the Delta wave and symptoms for 90 or more days (RR, 0.68; 95% CI, 0.46-1.02). In a sensitivity analysis using an alternative model, we considered the inclusion of history of hospitalization for COVID-19 instead of self-reported severity; hospitalized participants had a 38% higher risk of reporting 28 or more days of symptoms than those not hospitalized (RR, 1.38; 95% CI, 1.09-1.74).

**Table 2.** Multivariable Poisson Regression Models Reporting RRs of Longer-Term COVID-19 Symptoms Reported 28 or More Days or 90 or More Days After Symptom Onset

Variable	≥28 d (n = 1807)		≥90 d (n = 1726)	
	RR (95% CI)	*P* value	RR (95% CI)	*P* value
Unvaccinated prior to infection	1.39 (1.04-1.85)	.02	1.37 (0.88-2.13)	.17
Delta variant period^a^	0.92 (0.71-1.20)	.53	0.68 (0.46-1.02)	.07
Maximum symptom severity during acute illness				
None or mild	1 [Reference]	NA	1 [Reference]	NA
Moderate	1.80 (1.47-2.22)	.00	2.19 (1.62-2.96)	.00
Severe or critical	2.25 (1.80-2.81)	.00	2.88 (2.09-3.97)	.00
Days hospitalized^b^	1.02 (1.00-1.03)	.01	1.02 (1.00-1.03)	.03
Reported respiratory symptoms on enrollment form	1.14 (0.97-1.34)	.11	0.96 (0.76-1.20)	.70
Age group, y				
18-44	1 [Reference]	NA	1 [Reference]	NA
45-64	1.02 (0.81-1.30)	.85	1.10 (0.79-1.53)	.57
≥65	0.93 (0.62-1.41)	.74	1.20 (0.68-2.12)	.53
Sex				
Female	1.13 (0.97-1.31)	.11	1.20 (0.97-1.48)	.09
Male	1 [Reference]	NA	1 [Reference]	NA
Charlson Comorbidity Index score				
0	1 [Reference]	NA	1 [Reference]	NA
1-2	1.07 (0.83-1.38)	.59	0.85 (0.59-1.21)	.37
3-4	1.02 (0.68-1.53)	.91	0.80 (0.45-1.41)	.44
≥5	1.55 (1.01-2.37)	.046	1.25 (0.69-2.26)	.46
BMI category^c^				
Underweight or normal weight	1 [Reference]	NA	1 [Reference]	NA
Overweight	1.03 (0.84-1.26)	.77	0.98 (0.74-1.29)	.87
Obesity	0.95 (0.76-1.19)	.64	0.90 (0.66-1.23)	.51
Severe obesity	1.04 (0.81-1.35)	.74	1.12 (0.79-1.59)	.51

Abbreviations: BMI, body mass index (calculated as weight in kilograms divided by height in meters squared); NA, not applicable; RR, risk ratio.

^a^ July 1 to December 31, 2021.

^b^ The RRs reported are per day of hospitalization.

^c^ Underweight or normal weight was defined as less than 25; overweight, 25 to 29; obesity, 30 to 34; and severe obesity, 35 or higher.

Among the 1413 participants who were unvaccinated at the time of COVID-19 onset (77.1%), 67 of 537 (12.5%), 235 of 602 (39.0%), 485 of 836 (58.0%), 621 of 848 (73.2%), and 757 of 913 (82.9%) reported receiving at least 1 vaccination by the 1-, 3-, 6-, 9-, and 12-month post–symptom onset surveys, respectively. Vaccination after onset was associated with a 41% lower risk of reporting symptoms in only the 6-month survey (RR, 0.59; 95% CI, 0.40-0.89) (Table 3). Similar to the overall analysis, participants with greater initial illness symptom severity (moderate to severe) were more likely to report ongoing symptoms than those with mild symptoms.

**Table 3.** Multivariable Poisson Regression Models Considering Reporting Symptoms on the Survey as the Outcome, Including Only Participants Who Were Unvaccinated at Time of Symptom Onset With Surveys Filled Out Around the Specified Time Points

Variable	Time point^a^									
	1 Month (n = 537)		3 Months (n = 602)		6 Months (n = 836)		9 Months (n = 848)		12 Months (n = 913)	
	RR (95% CI)	*P* value	RR (95% CI)	*P* value	RR (95% CI)	*P* value	RR (95% CI)	*P* value	RR (95% CI)	*P* value
Vaccinated after infection, survey filled out >14 d after vaccination	0.68 (0.34-1.36)	.27	0.60 (0.35-1.05)	.08	0.59 (0.40-0.89)	.01	0.73 (0.46-1.17)	.19	0.68 (0.40-1.15)	.15
Delta variant period^b^	0.96 (0.49-1.86)	.89	0.88 (0.42-1.81)	.72	0.87 (0.43-1.73)	.68	0.43 (0.16-1.18)	.10	0.45 (0.14-1.45)	.18
Maximum symptom severity during acute illness										
None or mild	1 [Reference]	NA	1 [Reference]	NA	1 [Reference]	NA	1 [Reference]	NA	1 [Reference]	
Moderate	2.50 (1.44-4.35)	.001	1.55 (0.77-3.09)	.22	1.93 (1.11-3.34)	.02	1.90 (1.04-3.48)	.04	2.12 (1.16-3.88)	.01
Severe or critical	1.95 (1.10-3.45)	.02	2.62 (1.33-5.17)	.01	2.51 (1.42-4.43)	.002	2.72 (1.49-4.95)	.001	3.15 (1.73-5.74)	<.001
Age group, y										
18-44	1 [Reference]	NA	1 [Reference]	NA	1 [Reference]	NA	1 [Reference]	NA	1 [Reference]	NA
45-64	1.65 (1.09-2.49)	.02	0.77 (0.41-1.45)	.42	0.98 (0.63-1.54)	.93	0.86 (0.53-1.41)	.56	0.88 (0.54-1.43)	.61
≥65	1.51 (0.80-2.86)	.20	1.84 (0.85-3.99)	.12	1.53 (0.78-3.03)	.22	1.21 (0.57-2.57)	.62	1.71 (0.88-3.33)	.11
Sex										
Female	1.35 (0.92-1.98)	.13	1.37 (0.84-2.25)	.21	1.54 (1.03-2.30)	.03	1.16 (0.75-1.79)	.50	1.25 (0.81-1.93)	.30
Male	1 [Reference]	NA	1 [Reference]	NA	1 [Reference]	NA	1 [Reference]	NA	1 [Reference]	NA
BMI category^c^										
Underweight or normal weight	1 [Reference]	NA	1 [Reference]	NA	1 [Reference]	NA	1 [Reference]	NA	1 [Reference]	NA
Overweight	1.05 (0.59-1.87)	.86	1.33 (0.64-2.79)	.45	1.50 (0.81-2.79)	.20	1.03 (0.57-1.89)	.91	2.16 (1.04-4.51)	.04
Obesity	1.05 (0.57-1.94)	.87	1.37 (0.61-3.06)	.45	1.57 (0.82-3.01)	.18	0.93 (0.48-1.82)	.84	1.87 (0.86-4.07)	.11
Severe obesity	1.48 (0.81-2.71)	.20	1.46 (0.61-3.50)	.40	1.87 (0.94-3.72)	.07	1.31 (0.64-2.66)	.46	2.15 (0.95-4.88)	.07

Abbreviations: BMI, body mass index (calculated as weight in kilograms divided by height in meters squared); NA, not applicable; RR, risk ratio.

^a^ Time after symptom onset (or date of first positive SARS-CoV-2 test result if symptom onset date was not available): 1 month, 14 to 44 days; 3 months, 45 to 134 days; 6 months, 135 to 224 days; 9 months, 225 to 314 days; and 12 months, 315 to 404 days.

^b^ July 1 to December 31, 2021.

^c^ Underweight or normal weight was defined as less than 25; overweight, 25 to 29; obesity, 30 to 34; and severe obesity, 35 or higher.

**Figure 2.**
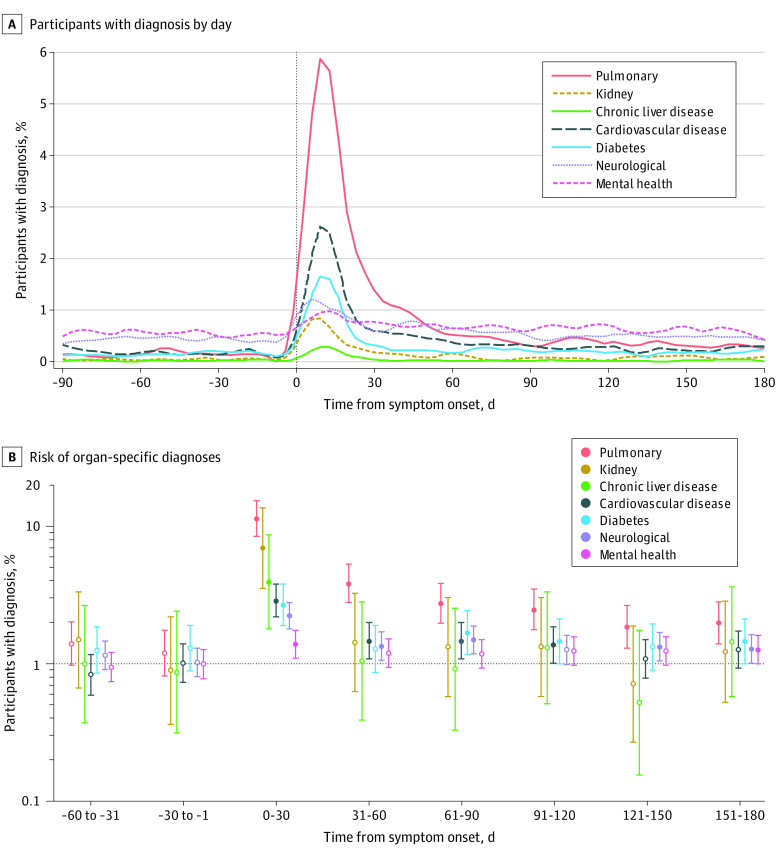
Health Care Encounters Before and After SARS-CoV-2 Infection Filled circles indicate *P* < .05; open circles, *P* > .05; and whiskers, 95% CIs.

In addition to survey data, we analyzed medical encounter data from participants’ medical records. Organ system–grouped diagnoses derived from medical encounters peaked in the month following COVID-19 symptom onset and decreased in frequency thereafter (Figure 2A). When considering organ-specific diagnoses as the outcome and using each participant’s experience with those diagnoses in the 61- to 90-day periods prior to COVID-19 symptom onset as the baseline, the risk of pulmonary, diabetes, neurological (eg, headache, loss of sense of taste and/or smell, or pain syndromes), and mental health–related medical encounters was elevated at 6 months after symptom onset compared with baseline (pulmonary: RR, 2.00; 95% CI, 1.40-2.84; diabetes: RR, 1.46; 95% CI, 1.00-2.13; neurological: RR, 1.29; 95% CI, 1.02-1.64; and mental health: RR, 1.28; 95% CI, 1.01-1.62) (Figure 2B). Those who were unvaccinated prior to infection were more likely to have encounters for some illness categories (pulmonary: RR, 1.72; 95% CI, 1.32-2.27; neurological: RR, 1.27; 95% CI, 1.00-1.59). Older age, higher BMI category, and COVID-19 hospitalization were consistently associated with these diagnosis categories and included in the model (eTable 3 in Supplement 1). In addition, women and those infected during the Delta wave were more likely to have medical encounters related to pulmonary, neurological, and mental health–related illnesses. Specific diagnoses and their frequencies are indicated in eFigure 2 in Supplement 1.

As a sensitivity analysis, we compared 657 people without survey data with those who were included in the study. The participants who did not respond to surveys were more likely to be younger, from racial and ethnic minority groups, male, or hospitalized (eTable 4 in Supplement 1).

## Discussion

In this longitudinal cohort of MHS beneficiaries, participants had a higher risk of health care encounters related to pulmonary, diabetes, neurological, and mental health diagnoses 6 months after infection compared with their pre–COVID-19 baseline, even after controlling for COVID-19 severity and other risk factors, and participants who reported more severe initial illnesses were more likely to have 28 or more days of symptoms. In addition, unvaccinated participants were at higher risk of 28 or more days of symptoms and of medical encounters associated with pulmonary and neurological diagnoses. Vaccination after infection was associated with a lower risk of reporting symptoms on the 6-month survey only.

From the surveys completed by participants, 39.7% of participants reported that their COVID-19 symptoms lasted 28 or more days, among whom half had symptoms that lasted 90 or more days, and 9.8% of the participants with a 6-month survey reported ongoing COVID-19 symptoms. These percentages with longer-term symptoms are somewhat higher than in a recent cross-sectional survey, which found that 14.7% of participants had continuing COVID-19 symptoms for 2 months or longer after symptom onset,^17^ and were lower than some of the other published percentages,^4,8,24^ potentially due to differences in the population characteristics (eg, the EPICC study population was younger, with a higher proportion of males, and 23% of infections were among fully vaccinated participants). In addition, some of the published studies were performed among hospitalized patients,^5,6,9,25^ whereas only 12.9% of the EPICC study participants included in this analysis were hospitalized due to COVID-19.

Participants who were vaccinated prior to COVID-19 onset were significantly less likely to report 28 or more days of illness, which is consistent with previously published findings.^16,17^ Other factors associated with longer-term symptoms that are identified in the literature, such as obesity, age, and female sex, were not associated with longer duration of symptoms when included in a multivariable model. A recent meta-analysis demonstrated that severity of COVID-19 (as measured by hospitalization) was not associated with risk of PCCs.^26^ In the EPICC study cohort, participants were at higher risk of reporting 28 or more days of symptoms if they were hospitalized during their initial infection or if they had higher self-reported symptom severity. Our findings may contribute to prognostic frameworks for advising those who may be more likely to develop PCCs.

Vaccination after onset was also associated with a lower risk of reporting symptoms at 6 months after symptom onset. The correlation between postinfection vaccination and mitigation of PCC risk has been noted,^15,27^ but further studies should confirm this and also examine possible mechanisms that may explain this statistical association. The observations of postinfection vaccination benefit may reflect enhanced clearance of persistent virus^28^ or nonspecific immunomodulation, which may alter possible inflammatory drivers of PCC symptoms.

We augmented our patient-reported outcome survey findings with use of medical encounter data to further define the risk of PCCs in this study population, adjusting for prior health care use. SARS-CoV-2 infection was associated with a large increase in the frequency of clinical encounters in the first month after symptom onset across multiple organ systems and did not return to baseline for the pulmonary, diabetes, and neurology-related diagnoses or for mental health medical encounters at 6 months. These findings indicate that COVID-19 illness may be associated with a higher rate of seeking medical attention for those symptoms up to 6 months after infection, even after adjusting for pre–COVID-19 health care diagnoses. We also noted a high frequency of medical encounters for other organ system diagnoses up to several months after symptom onset. Taken together, this represents a substantive burden of medical care utilization after COVID-19, even after adjusting for health care use prior to COVID-19 infection.

### Strengths and Limitations

There are several strengths to this study, including use of the MDR, which allows individuals to act as their own controls longitudinally when considering medical encounters and organ system diagnoses and which complements the patient-reported outcomes. Our evaluation of independent factors associated with risk of persistent symptoms was strengthened by the availability of important confounders measured in the EPICC study. Another strength of our study was the enrollment of a relatively young, healthy study population, complementing other PCC studies.^2,6,25^ In addition, the long follow-up time allowed for a substantial sample size to describe the burden of PCC and symptoms up to 6 months.

There were also several limitations to this study. First, participants volunteered for the EPICC study, which initially recruited primarily among those seeking testing and treatment for SARS-CoV-2 infection and therefore has a comparatively higher percentage of symptomatic participants. Second, the persistent symptoms and ongoing diagnoses in this analysis can be nonspecific to COVID-19 and may be frequent in those without SARS-CoV-2 infection,^29^ and our lack of a control group may bias the conclusions. Recurring diagnoses might have been identified as a result of health care sought for SARS-CoV-2 infection but not from the infection itself. In addition, 657 participants were missing survey data. When compared with those who were included in the study, the participants who did not respond to surveys were more likely to be younger, from racial and ethnic minority groups, male, or hospitalized. Also, to minimize follow-up survey data missingness, we focused on 6-month postinfection data, and we excluded those with enrollment in 2022 during the Omicron wave. Future studies will be important to improve understanding of the heterogeneity of PCC risk, including Omicron-related PCC risk, reinfections, and longer-term PCC risk and risk factors beyond 6 months.

## Conclusions

In this cohort study, more severe acute COVID-19 illness, a higher CCI score, and being unvaccinated prior to infection were associated with a higher risk of reporting 28 or more days of COVID-19 symptoms, and participants with COVID-19 were more likely to seek medical care for diabetes, pulmonary, neurology, and mental health–related diagnoses for at least 6 months after COVID-19 onset compared with their pre–COVID-19 health care use patterns. Our findings suggest that PCCs present a major burden to patients and the health care systems that treat them. Our observational data offer further evidence that postinfection vaccination may mitigate PCCs. Taken together, these findings may inform the risk-benefit ratio of COVID-19 vaccination policy.

## Notes

Supplement 1. **eFigure 1.** Flow Chart of Inclusion in the Analyses of EPICC Cohort Participants **eFigure 2.** Specific Diagnoses in EPICC Participants’ Health Records From 90 Days Prior to 180 After SARS-CoV-2 Diagnosis **eTable 1.** Schedule of Specimen and Data Collection for Participants Enrolled in the EPICC Cohort Study **eTable 2.** *ICD-10* Categories Used to Categorize Medical Diagnoses **eTable 3.** Risk of Organ-Specific Diagnoses Derived From Generalized Linear Models Run Using Each Organ System Category as the Outcome **eTable 4.** Comparison of Those Who Were and Were Not Included in the Analysis Based on Availability of Survey and Military Health System Repository Data

Supplement 2. **Nonauthor Collaborators.** Epidemiology, Immunology, and Clinical Characteristics of Emerging Infectious Diseases With Pandemic Potential (EPICC) COVID-19 Cohort Study Group

Supplement 3.
Data Sharing Statement
