# Error in Figure 2B

**DOI:** 10.1001/jamanetworkopen.2023.0734

**Published:** 2023-02-09

In the Original Investigation titled “Persistent COVID-19 Symptoms at 6 Months After Onset and the Role of Vaccination Before or After SARS-CoV-2 Infection,”^1^ published January 18, 2023, the y*-*axis label in Figure 2B should be “risk ratio” instead of “participants with diagnosis, %.” This article has been corrected.^1^
