# Supplementary material for: Persistent COVID-19 Symptoms at 6 Months After Onset and the Role of Vaccination Before or After SARS-CoV-2 Infection
Source: JAMA Netw Open. 2023 Jan 18;6(1):e2251360. doi: 10.1001/jamanetworkopen.2022.51360 (PMC9857077; doi:10.1001/jamanetworkopen.2022.51360)
Supplement: Supplement 1. — eFigure 1. Flow Chart of Inclusion in the Analyses of EPICC Cohort Participants eFigure 2. Specific Diagnoses in EPICC Participants’ Health Records From 90 Days Prior to 180 After SARS-CoV-2 Diagnosis eTable 1. Schedule of Specimen and Data Collection for Participants Enrolled in the EPICC Cohort Study eTable 2. ICD-10 Categories Used to Categorize Medical Diagnoses eTable 3. Risk of Organ-Specific Diagnoses Derived From Generalized Linear Models Run Using Each Organ System Category as the Outcome eTable 4. Comparison of Those Who Were and Were Not Included in the Analysis Based on Availability of Survey and Military Health System Repository Data [file jamanetwopen-e2251360-s001.pdf]

Supplementary Online Content

Richard SA, Pollett SD, Fries AC, et al; Epidemiology, Immunology, and Clinical Characteristics of Emerging Infectious Diseases With Pandemic Potential (EPICC) COVID-19 Cohort Study Group. Persistent COVID-19 symptoms at 6 months after onset and the role of vaccination before or after SARS-CoV-2 infection. *JAMA Netw Open*. 2023;6(1):e2251360. doi:10.1001/jamanetworkopen.2022.51360

eFigure 1. Flow Chart of Inclusion in the Analyses of EPICC Cohort Participants

eFigure 2. Specific Diagnoses in EPICC Participants' Health Records From 90 Days Prior to 180 After SARS-CoV-2 Diagnosis

eTable 1. Schedule of Specimen and Data Collection for Participants Enrolled in the EPICC Cohort Study

eTable 2. *ICD-10* Categories Used to Categorize Medical Diagnoses

eTable 3. Risk of Organ-Specific Diagnoses Derived From Generalized Linear Models Run Using Each Organ System Category as the Outcome

eTable 4. Comparison of Those Who Were and Were Not Included in the Analysis Based on Availability of Survey and Military Health System Repository Data

This supplementary material has been provided by the authors to give readers additional information about their work.

© 2023 Richard SA et al. *JAMA Network Open*.

eFigure 1. Flow chart of inclusion in the analyses of EPICC cohort participants.

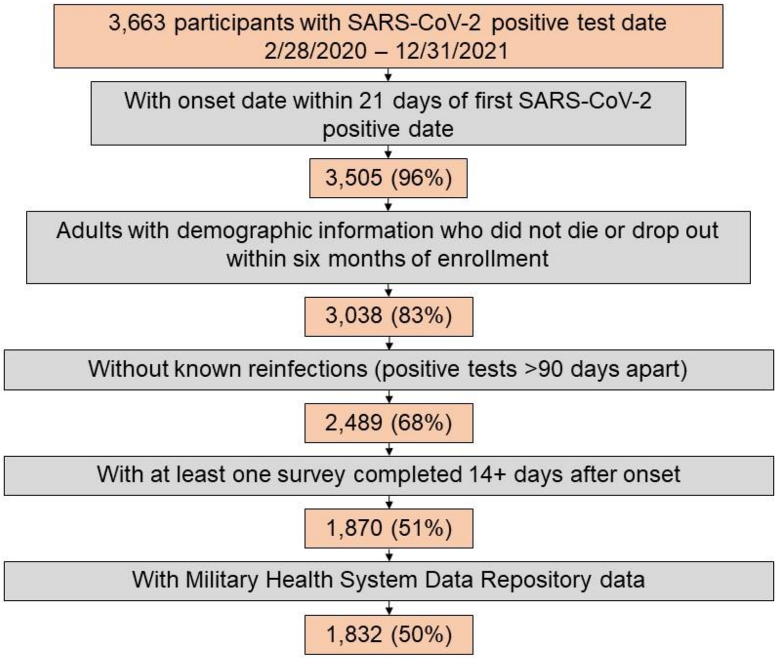

© 2023 Richard SA et al. *JAMA Network Open*.

eFigure 2. Specific diagnoses in EPICC participants' health records from 90 days prior to 180 after SARS-CoV-2 diagnosis.

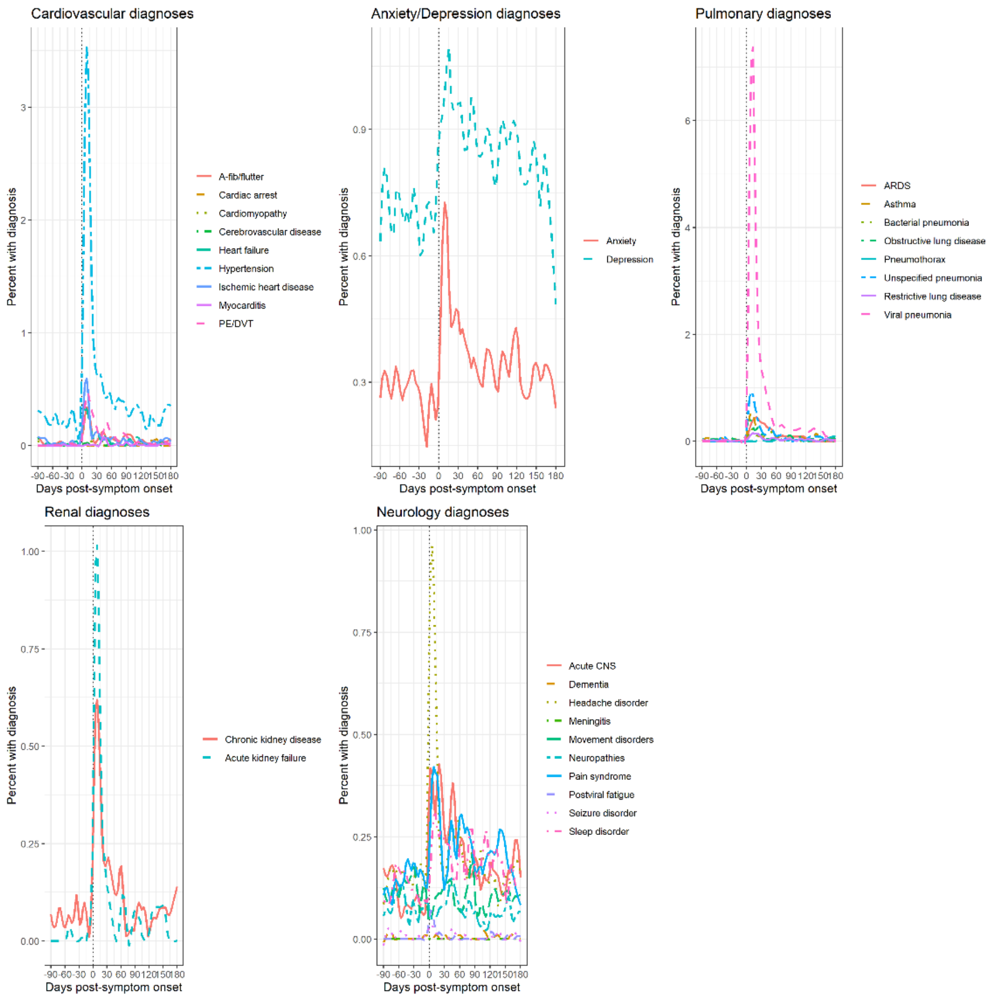

© 2023 Richard SA et al. *JAMA Network Open*.

eTable 1. Schedule of specimen and data collection for participants enrolled in the EPICC cohort study (H=hospitalized Military Treatment Facility (MTF) participant; O=outpatient MTF participant; I=internet-based participant)

	Day 0	Day 3	Day 7	Weekly	Day 14	Day 28	3 months	6 months	9 months	12 months
Virology										
Nasal-throat-and/or oral swab[+]	H+O	H	H	H	H+O					
Immunology										
Blood[‡]	H+O+I	H	H+O	H	H+O	H+O+I		H+O+I		H+O+1
Clinical characteristics										
FLU-PRO plus symptom diary*	H+O									
Survey	H+O+I					H+O+I	H+O+I	H+O+I	H+O+I	H+O+I

[+] Swab collected for outpatients or after hospital discharge (for inpatients) may be performed using self-collected nasal or staff-collected NP/OP specimens.

[‡]Blood collection for outpatients or after hospital discharge (for inpatients) may be performed using an at-home volumetric absorptive microsampling (VAMS) device.

*FLU-PRO plus symptom diary collected daily for 14 days

© 2023 Richard SA et al. *JAMA Network Open*.

eTable 2. *ICD-10* categories used to categorize medical diagnoses.

Group	Dx	Included Codes (and subcodes unless otherwise noted)	Additional details
Cardiovascular Disease	Hypertension	I10-I16 Hypertensive diseases<br>H35.03x Hypertensive retinopathy<br>I67.4 Hypertensive encephalopathy	I10 Essential (primary) hypertension<br>I11 Hypertensive heart disease<br>I12 Hypertensive chronic kidney disease<br>I13 Hypertensive heart and chronic kidney disease<br>I15 Secondary hypertension<br>I16 Hypertensive crisis<br>H35.031-H35.032-H35.033-H35.039 Hypertensive retinopathy<br>I67.4 Hypertensive encephalopathy
Cardiovascular Disease	Ischemic Heart Disease	I20-I25 Ischemic heart diseases	I20 Angina pectoris<br>I21 Acute myocardial infarction<br>I22 Subsequent ST elev. (STEMI) and non-ST elev. (NSTEMI) myocardial infarction<br>I23 Certain current complications following ST elevation (STEMI) and non-ST elevation (NSTEMI) myocardial infarction (within the 28 day period)<br>I24 Other acute ischemic heart diseases<br>I25 Chronic ischemic heart disease
Cardiovascular Disease	Pulmonary Embolism and Deep vein thrombosis	G08 Intracranial and intraspinal thrombosis/phlebitis<br>I26 Pulmonary embolism<br>I80 Phlebitis and thrombophlebitis<br>I82 Other venous embolism and thrombosis	
Cardiovascular Disease	Myocarditis	I40 Acute myocarditis<br>I51.4 Myocarditis-unspecified	
Cardiovascular Disease	Cardiomyopathy	I42 Cardiomyopathy	
Cardiovascular Disease	Atrial fibrillation / flutter	I48 Atrial fibrillation and flutter	

© 2023 Richard SA et al. *JAMA Network Open*.

Cardiovascular Disease	Cardiac Arrest and Ventricular Tachycardia	I46 Cardiac arrest<br>I47.2 Ventricular tachycardia	
Cardiovascular Disease	Heart Failure	I50 Heart failure<br>R57.0 Cardiogenic shock	
Cardiovascular Disease	Cerebrovascular Disease	I60-I69 Cerebrovascular diseases<br>G45 Transient cerebral ischemic attacks and related syndromes<br>G46 Vascular syndromes of brain in cerebrovascular diseases<br>H34 Retinal vascular occlusions	I60 Nontraumatic subarachnoid hemorrhage<br>I61 Nontraumatic intracerebral hemorrhage<br>I62 Other and unspecified nontraumatic intracranial hemorrhage<br>I63 Cerebral infarction<br>I65 Occlusion and stenosis of precerebral arteries-not resulting in cerebral infarction<br>I66 Occlusion and stenosis of cerebral arteries-not resulting in cerebral infarction<br>I67 Other cerebrovascular diseases<br>I68 Cerebrovascular disorders in diseases classified elsewhere<br>I69 Sequelae of cerebrovascular disease
Pulmonary	Pneumonia-Viral	J12 Viral pneumonia-not elsewhere classified	
Pulmonary	Pneumonia-Bacterial	J13 Pneumonia due to Streptococcus pneumoniae<br>J14 Pneumonia due to Hemophilus influenzae<br>J15 Bacterial pneumonia-not elsewhere classified	
Pulmonary	Pneumonia-NOS	J16 Pneumonia due to other infectious organisms-not elsewhere classified<br>J17 Pneumonia in diseases classified elsewhere<br>J18 Pneumonia-unspecified organism	
Pulmonary	Asthma	J45 Asthma	
Pulmonary	Obstructive Lung Disease	J41-J44-J47 Chronic lower respiratory diseases	[note: J40 excluded as acute/chronic not specified; n=3]<br>J41 Simple and mucopurulent chronic bronchitis<br>J42 Unspecified chronic bronchitis<br>J43 Emphysema<br>J44 Other chronic obstructive pulmonary disease<br>J47 Bronchiectasis<br>[note: J98.8 Other specified respiratory disorders not included due to acute viral infection; n=7]

© 2023 Richard SA et al. *JAMA Network Open*.

Pulmonary	Restrictive Lung Disease	J84 Other interstitial pulmonary diseases<br>J98.4 Other disorders of lung	
Pulmonary	Pneumothorax	J93 Pneumothorax and air leak	
Pulmonary	ARDS	J80 Acute respiratory distress syndrome	
Neurology	Meningitis and Encephalitis	G00-G05 Meningitis and Encephalitis	G00 Bacterial meningitis-not elsewhere classified<br>G01 Meningitis in bacterial diseases classified elsewhere<br>G02 Meningitis in other infectious and parasitic diseases classified elsewhere<br>G03 Meningitis due to other and unspecified causes<br>G04 Encephalitis-myelitis and encephalomyelitis<br>G05 Encephalitis-myelitis and encephalomyelitis in diseases classified elsewhere
Neurology	Seizure Disorder	G40 Epilepsy and recurrent seizures	
Neurology	Headache Disorder	G43 Migraine<br>G44 Other headache syndromes	
Neurology	Sleep Disorder	G47 Sleep disorders (except G47.3)	G47.3 Sleep apnea (n=89) excluded
Neurology	Pain Syndrome NOS	G89 Pain-not elsewhere classified	
Neurology	Acute CNS signs and symptoms (not intoxication/ withdrawal)	F05 Delirium due to known physiological condition<br>F09 Unspecified mental disorder due to known physiological condition<br>R40-R46 Symptoms and signs involving cognition-perception-emotional state and behavior	F05 Delirium due to known physiological condition<br>F09 Unspecified mental disorder due to known physiological condition<br>R40 Somnolence-stupor and coma<br>R41 Other symptoms and signs involving cognitive functions and awareness<br>R42 Dizziness and giddiness<br>R43 Disturbances of smell and taste<br>R44 Other symptoms and signs involving general sensations and perceptions<br>R45 Symptoms and signs involving emotional state<br>R46 Symptoms and signs involving appearance and behavior
Neurology	Postviral Fatigue or Encephalopathy	G93.3 Postviral fatigue syndrome<br>G93.4 Other and unspecified encephalopathy	

© 2023 Richard SA et al. *JAMA Network Open*.

Neurology	Dementia	F01 Vascular dementia<br>F02 Dementia in other diseases classified elsewhere<br>F03 Unspecified dementia<br>G30 Alzheimer's disease<br>G31 Other degenerative diseases of CNS	
Neurology	Movement Disorders	G20-G26 Extrapyramidal and movement disorders	G20 Parkinson's disease<br>G21 Secondary parkinsonism<br>G23 Other degenerative diseases of basal ganglia<br>G24 Dystonia<br>G25 Other extrapyramidal and movement disorders<br>G26 Extrapyramidal and movement disorders in diseases classified elsewhere
Neurology	Neuropathies	G50-G65 Polyneuropathies and other disorders of the peripheral nervous system	G50 Disorders of trigeminal nerve<br>G51 Facial nerve disorders<br>G52 Disorders of other cranial nerves<br>G53 Cranial nerve disorders in diseases classified elsewhere<br>G54 Nerve root and plexus disorders<br>G55 Nerve root and plexus compressions in diseases classified elsewhere<br>G56 Mononeuropathies of upper limb<br>G57 Mononeuropathies of lower limb<br>G58 Other mononeuropathies<br>G59 Mononeuropathy in diseases classified elsewhere<br>G60 Hereditary and idiopathic neuropathy<br>G61 Inflammatory polyneuropathy<br>G62 Other and unspecified polyneuropathies<br>G63 Polyneuropathy in diseases classified elsewhere<br>G64 Other disorders of peripheral nervous system<br>G65 Sequelae of inflammatory and toxic polyneuropathies
Mental Health	Depression and Mood Disorders	F30-F39 Mood [affective] disorders	F30 Manic episode<br>F31 Bipolar disorder<br>F32 Major depressive disorder-single episode<br>F33 Major depressive disorder-recurrent

© 2023 Richard SA et al. *JAMA Network Open*.

			F34 Persistent mood [affective] disorders<br>F39 Unspecified mood [affective] disorder
Mental Health	Anxiety and Somatoform Disorders	F41-F48 Anxiety-dissociative-stress-related-and somatoform disorders	F41 Other anxiety disorders<br>F42 Obsessive-compulsive disorder<br>F43 Reaction to severe stress-and adjustment disorders<br>F44 Dissociative and conversion disorders<br>F45 Somatoform disorders<br>F48 Other nonpsychotic mental disorders
Endocrine	Diabetes	E08-E13 Diabetes mellitus	E08 Diabetes mellitus due to underlying condition<br>E09 Drug or chemical induced diabetes mellitus<br>E10 Type 1 diabetes mellitus<br>E11 Type 2 diabetes mellitus<br>E13 Other specified diabetes mellitus
Liver	Liver Disease-Chronic	K70-K77 Diseases of liver<br>Z94.4 Liver transplant status	K70 Alcoholic liver disease<br>K71 Toxic liver disease<br>K72 Hepatic failure-not elsewhere classified<br>K73 Chronic hepatitis-not elsewhere classified<br>K74 Fibrosis and cirrhosis of liver<br>K75 Other inflammatory liver diseases<br>K76 Other diseases of liver<br>K77 Liver disorders in diseases classified elsewhere<br>Z94.4 Liver transplant status
Renal	Acute Kidney Failure	N17 Acute kidney failure	
Renal	Chronic Kidney Disease	I12 Hypertensive chronic kidney disease<br>I13 Hypertensive heart and chronic kidney disease<br>N18 Chronic kidney disease (CKD)<br>N19 Unspecified kidney failure<br>Z49 Encounter for care involving renal dialysis<br>Z94.0 Kidney transplant status<br>Z99.2 Dependence on renal dialysis	
Other	Bacteremia	R78.81 Bacteremia	

© 2023 Richard SA et al. *JAMA Network Open*.

Other	DIC/ Coagulopathy	D65 Disseminated intravascular coagulation [defibrination syndrome]<br>D68 Other coagulation defects<br>D69.3 Immune thrombocytopenic purpura<br>D69.8 Other specified hemorrhagic conditions<br>D69.9 Hemorrhagic condition-unspecified	

© 2023 Richard SA et al. *JAMA Network Open*.

eTable 3. Risk of organ-specific diagnoses derived from generalized linear models run using each organ system category as the outcome. The models included time in 30-day periods around symptom onset, all variables listed in table, as well as a random effect for the participant.

	Pulmonary		Renal		Liver		Cardiovascular disease		Diabetes		Neurology		Mental health	
Variable	Risk ratio	p-value	Risk ratio	p-value	Risk ratio	p-value	Risk ratio	p-value	Risk ratio	p-value	Risk ratio	p-value	Risk ratio	p-value
61-90 days pre-onset	Ref		Ref		Ref		Ref		Ref		Ref		Ref	
31-60 days pre-onset	1.41 (0.98-2.03)	0.07	1.50 (0.67-3.34)	0.32	1.00 (0.38-2.66)	1.00	0.84 (0.59-1.17)	0.30	1.26 (0.86-1.86)	0.24	1.16 (0.92-1.47)	0.21	0.95 (0.74-1.21)	0.66
1-30 days pre-onset	1.20 (0.82-1.76)	0.34	0.90 (0.37-2.21)	0.82	0.88 (0.32-2.41)	0.80	1.01 (0.73-1.40)	0.93	1.30 (0.89-1.92)	0.18	1.03 (0.81-1.31)	0.81	1.00 (0.79-1.27)	1.00
0-30 days post-onset	11.33 (8.42-15.26)	0.00	6.96 (3.56-13.62)	0.00	3.95 (1.80-8.66)	0.00	2.89 (2.20-3.79)	0.00	2.69 (1.91-3.79)	0.00	2.24 (1.81-2.78)	0.00	1.40 (1.11-1.77)	0.00
31-60 days post-onset	3.84 (2.78-5.29)	0.00	1.43 (0.63-3.25)	0.39	1.05 (0.39-2.82)	0.92	1.48 (1.09-2.00)	0.01	1.29 (0.88-1.90)	0.20	1.35 (1.07-1.71)	0.01	1.20 (0.95-1.53)	0.13
61-90 days post-onset	2.76 (1.97-3.86)	0.00	1.33 (0.58-3.05)	0.50	0.92 (0.33-2.55)	0.87	1.48 (1.09-2.00)	0.01	1.70 (1.18-2.45)	0.00	1.50 (1.19-1.90)	0.00	1.19 (0.93-1.51)	0.16
91-120 days post-onset	2.49 (1.77-3.50)	0.00	1.33 (0.58-3.05)	0.50	1.32 (0.52-3.35)	0.57	1.38 (1.02-1.88)	0.04	1.46 (1.00-2.13)	0.05	1.27 (1.00-1.61)	0.05	1.25 (0.98-1.58)	0.07
121-150 days post-onset	1.86 (1.30-2.66)	0.00	0.72 (0.27-1.89)	0.50	0.53 (0.16-1.75)	0.30	1.09 (0.79-1.51)	0.58	1.33 (0.91-1.96)	0.14	1.34 (1.06-1.70)	0.02	1.24 (0.98-1.57)	0.08
151-180 days post-onset	2.00 (1.40-2.84)	0.00	1.23 (0.53-2.86)	0.63	1.45 (0.58-3.62)	0.43	1.27 (0.93-1.74)	0.13	1.46 (1.00-2.13)	0.05	1.29 (1.02-1.64)	0.04	1.28 (1.01-1.62)	0.04
Unvaccinated prior to infection	1.72 (1.32-2.27)	0.00	1.30 (0.46-3.70)	0.62	2.22 (0.52-10.0)	0.28	0.99 (0.69-1.41)	0.95	0.90 (0.54-1.52)	0.70	1.27 (1.00-1.59)	0.05	1.11 (0.86-1.43)	0.43
Delta variant period (July 1, 2021 to December 31, 2021)	1.28 (1.01-1.61)	0.04	0.66 (0.25-1.75)	0.41	0.57 (0.20-1.59)	0.28	1.08 (0.76-1.52)	0.68	0.57 (0.32-1.02)	0.06	1.36 (1.06-1.73)	0.02	1.53 (1.07-2.19)	0.02
Hospitalized for COVID-19	3.26 (2.67-3.97)	0.00	9.60 (4.76-19.34)	0.00	4.31 (2.21-8.41)	0.00	3.38 (2.53-4.51)	0.00	4.08 (2.53-6.57)	0.00	1.62 (1.21-2.16)	0.00	1.71 (1.07-2.72)	0.03
Age group: 18-44	Ref		Ref		Ref		Ref		Ref		Ref		Ref	
Age group: 45-64	1.39 (1.16-1.67)	0.00	5.03 (2.28-11.09)	0.00	3.67 (1.88-7.16)	0.00	5.30 (3.98-7.05)	0.00	5.21 (3.29-8.25)	0.00	1.17 (0.94-1.47)	0.17	0.78 (0.55-1.12)	0.18
Age group: 65+	2.61 (2.02-3.38)	0.00	30.15 (11.99-75.84)	0.00	2.23 (0.79-6.24)	0.13	14.70 (9.97-21.68)	0.00	20.49 (10.91-38.50)	0.00	1.09 (0.73-1.61)	0.69	0.45 (0.22-0.89)	0.02
Sex: Female	1.26 (1.07-1.47)	0.00	0.80 (0.42-1.55)	0.51	1.27 (0.70-2.29)	0.43	0.98 (0.77-1.26)	0.90	3.40 (2.26-5.10)	0.00	1.43 (1.17-1.73)	0.00	2.90 (2.13-3.94)	0.00
BMI category: Underweight/normal	Ref		Ref		Ref		Ref		Ref		Ref		Ref	

© 2023 Richard SA et al. *JAMA Network Open*.

BMI category: Overweight	1.13 (0.90-1.41)	0.30	2.25 (0.72-7.01)	0.16	0.88 (0.32-2.42)	0.81	1.41 (0.96-2.08)	0.08	1.32 (0.71-2.46)	0.38	1.07 (0.82-1.39)	0.62	1.89 (1.24-2.87)	0.00
BMI category: Obese	1.52 (1.20-1.93)	0.00	2.81 (0.88-8.96)	0.08	2.05 (0.77-5.45)	0.15	2.06 (1.38-3.09)	0.00	2.97 (1.57-5.62)	0.00	1.33 (1.00-1.77)	0.05	2.61 (1.65-4.13)	0.00
BMI category: Severely obese	1.61 (1.24, 2.10)	0.00	2.03 (0.59, 6.94)	0.26	3.06 (1.13, 8.24)	0.03	3.02 (1.97, 4.64)	0.00	3.99 (2.03, 7.81)	0.00	1.34 (0.96, 1.88)	0.09	2.41 (1.41, 4.11)	0.00

© 2023 Richard SA et al. *JAMA Network Open*.

eTable 4. Comparison of those who were and were not included in the analysis based on availability of survey and Military Health System Data Repository data. Statistical comparisons are Pearson's Chi squared tests.

	Not included (N=657)	Included (N=1832)	Total (N=2489)	p value
**Age group**				< 0.001
18-44	486 (74.0%)	1226 (66.9%)	1712 (68.8%)	
45-64	123 (18.7%)	487 (26.6%)	610 (24.5%)	
65+	48 (7.3%)	119 (6.5%)	167 (6.7%)	
**Male**				0.010
Female	219 (33.3%)	714 (39.0%)	933 (37.5%)	
Male	438 (66.7%)	1118 (61.0%)	1556 (62.5%)	
**Race/ethnicity**				0.003
Asian	33 (5.0%)	78 (4.3%)	111 (4.5%)	
Black	93 (14.2%)	206 (11.2%)	299 (12.0%)	
Hispanic or Latino	168 (25.6%)	384 (21.0%)	552 (22.2%)	
Other	38 (5.8%)	154 (8.4%)	192 (7.7%)	
White	325 (49.5%)	1010 (55.1%)	1335 (53.6%)	
**BMI category**				0.650
Under/normal weight	157 (23.9%)	399 (21.8%)	556 (22.3%)	
Overweight	269 (40.9%)	760 (41.5%)	1029 (41.3%)	
Obese	153 (23.3%)	432 (23.6%)	585 (23.5%)	
Severely obese	78 (11.9%)	241 (13.2%)	319 (12.8%)	
**Charlson Comorbidity Index category**				0.094

© 2023 Richard SA et al. *JAMA Network Open*.

0	479 (72.9%)	1290 (70.4%)	1769 (71.1%)	
1-2	115 (17.5%)	373 (20.4%)	488 (19.6%)	
3-4	29 (4.4%)	102 (5.6%)	131 (5.3%)	
5+	34 (5.2%)	67 (3.7%)	101 (4.1%)	
**Military status**				0.159
Active duty	431 (65.6%)	1126 (61.5%)	1557 (62.6%)	
Dependent	126 (19.2%)	383 (20.9%)	509 (20.4%)	
Retired military	100 (15.2%)	323 (17.6%)	423 (17.0%)	
**Infected during Delta period (7/1/21-12/31/21)**				0.574
Pre-Delta	476 (72.5%)	1348 (73.6%)	1824 (73.3%)	
Delta	181 (27.5%)	484 (26.4%)	665 (26.7%)	
**Hospitalization status**				0.005
Hospitalized	114 (17.4%)	236 (12.9%)	350 (14.1%)	
Outpatient	543 (82.6%)	1596 (87.1%)	2139 (85.9%)	

© 2023 Richard SA et al. *JAMA Network Open*.
