# Supplementary material for: Persistent COVID-19 Symptoms at 6 Months After Onset and the Role of Vaccination Before or After SARS-CoV-2 Infection
Source: JAMA Netw Open. 2023 Jan 18;6(1):e2251360. doi: 10.1001/jamanetworkopen.2022.51360 (PMC9857077; doi:10.1001/jamanetworkopen.2022.51360)
Supplement: Supplement 2. — Nonauthor Collaborators. Epidemiology, Immunology, and Clinical Characteristics of Emerging Infectious Diseases With Pandemic Potential (EPICC) COVID-19 Cohort Study Group [file jamanetwopen-e2251360-s002.pdf]

Supplemental Online Content: Nonauthor Collaborators
*First name, last name, and suffix (if applicable) are required and will appear in PubMed.

***Group Name(s): Epidemiology, Immunology, and Clinical Characteristics of Emerging Infectious Diseases With Pandemic Potential (EPICC) COVID-19 Cohort Study Group**

***First Name and Middle Initial(s)**	***Last Name**	***Suffix (eg, Jr, III)**	Academic Degrees	Institution	Location (city, state/province, country)	Role or Contribution, eg, chair, principal investigator	Group (if more than 1 Group listed in the byline) and/or Subgroup (eg, Steering Committee)
Terry	Merritt			Brooke Army Medical Center	Fort Sam Houston, TX		EPICC COVID-19 Cohort Study Group
Susan	Chambers			Madigan Army Medical Center	Joint Base Lewis McChord, WA		EPICC COVID-19 Cohort Study Group
Cristin	Mount			Madigan Army Medical Center	Joint Base Lewis McChord, WA		EPICC COVID-19 Cohort Study Group
Michael	Stein			Madigan Army Medical Center	Joint Base Lewis McChord, WA		EPICC COVID-19 Cohort Study Group
Catherine	Uyehara			Tripler Army Medical Center	Honolulu, HI		EPICC COVID-19 Cohort Study Group
Heidi	Adams			Uniformed Services University of the Health Sciences	Bethesda, MD		EPICC COVID-19 Cohort Study Group
Patrick	Hickey			Uniformed Services University of the Health Sciences	Bethesda, MD		EPICC COVID-19 Cohort Study Group
Edward	Parmelee			Uniformed Services University of the Health Sciences	Bethesda, MD		EPICC COVID-19 Cohort Study Group
Mark	Fritschlanski			Uniformed Services University of the Health Sciences	Bethesda, MD		EPICC COVID-19 Cohort Study Group
Tim	Chao			United States Air Force School of Aerospace Medicine	Dayton, OH		EPICC COVID-19 Cohort Study Group
Richard	Chapleau			United States Air Force School of Aerospace Medicine	Dayton, OH		EPICC COVID-19 Cohort Study Group
Monica	Christian			United States Air Force School of Aerospace Medicine	Dayton, OH		EPICC COVID-19 Cohort Study Group
Kelsey	Lanter			United States Air Force School of Aerospace Medicine	Dayton, OH		EPICC COVID-19 Cohort Study Group
Jennifer	Meyer			United States Air Force School of Aerospace Medicine	Dayton, OH		EPICC COVID-19 Cohort Study Group

*First name, last name, and suffix (if applicable) are required and will appear in PubMed.

***First Name and Middle Initial(s)**	***Last Name**	***Suffix (eg, Jr, III)**	Academic Degrees	Institution	Location (city, state/province, country)	Role or Contribution, eg, chair, principal investigator	Group (if more than 1 Group listed in the byline) and/or Subgroup (eg, Steering Committee)
John	Iskander			United States Coast Guard	Washington, DC		EPICC COVID-19 Cohort Study Group
Kathryn	Lago			Womack Army Medical Center	Fort Bragg, NC		EPICC COVID-19 Cohort Study Group
