# Supplementary material for: Persistent COVID-19 Symptoms at 6 Months After Onset and the Role of Vaccination Before or After SARS-CoV-2 Infection
Source: JAMA Netw Open. 2023 Jan 18;6(1):e2251360. doi: 10.1001/jamanetworkopen.2022.51360 (PMC9857077; doi:10.1001/jamanetworkopen.2022.51360)
Supplement: Supplement 3. — Data Sharing Statement [file jamanetwopen-e2251360-s003.pdf]

Data Sharing Statement

Richard. Persistent COVID-19 Symptoms at 6 Months After Onset and the Role of Vaccination Before or After SARS-CoV-2 Infection. *JAMA Netw Open*. Published January 18, 2023. doi:10.1001/jamanetworkopen.2022.51360

Data
Data available: No
